# Effectiveness, safety and acceptability of no‐test medical abortion (termination of pregnancy) provided via telemedicine: a national cohort study

**DOI:** 10.1111/1471-0528.16668

**Published:** 2021-03-24

**Authors:** ARA Aiken, PA Lohr, J Lord, N Ghosh, J Starling

**Affiliations:** ^1^ LBJ School of Public Affairs University of Texas at Austin Austin TX USA; ^2^ British Pregnancy Advisory Service Stratford upon Avon UK; ^3^ MSI Reproductive Choices London UK; ^4^ National Unplanned Pregnancy Advisory Service (NUPAS) Birmingham UK; ^5^ Mathematica Policy Research Cambridge MA USA

**Keywords:** Abortion, induced [E04.520.050], ambulatory care facilities [N02.278.035], health planning [N03.349], mifepristone [D04.210.500.365.415.580], misoprostol [D23.469.700.660.500], pregnancy complications [C13.703], telemedicine [N04.590.374.800], termination of pregnancy

## Abstract

**Objective:**

To compare outcomes before and after implementation of medical abortion (termination of pregnancy) without ultrasound via telemedicine.

**Design:**

Cohort analysis.

**Setting:**

The three main abortion providers.

**Population or sample:**

Medical abortions at home at ≤69 days’ gestation in two cohorts: traditional model (in‐person with ultrasound, *n* = 22 158) from January to March 2020 versus telemedicine‐hybrid model (either in person or via telemedicine without ultrasound, *n* = 29 984, of whom 18 435 had no‐test telemedicine) between April and June 2020. Sample (*n* = 52 142) comprises 85% of all medical abortions provided nationally.

**Methods:**

Data from electronic records and incident databases were used to compare outcomes between cohorts, adjusted for baseline differences.

**Main outcome measures:**

Treatment success, serious adverse events, waiting times, gestation at treatment, acceptability.

**Results:**

Mean waiting time from referral to treatment was 4.2 days shorter in the telemedicine‐hybrid model and more abortions were provided at ≤6 weeks’ gestation (40% versus 25%, *P* < 0.001). Treatment success (98.8% versus 98.2%, *P* > 0.999), serious adverse events (0.02% versus 0.04%, *P* = 0.557) and incidence of ectopic pregnancy (0.2% versus 0.2%, *P* = 0.796) were not different between models. In the telemedicine‐hybrid model, 0.04% were estimated to be over 10 weeks’ gestation at the time of the abortion; all were completed safely at home. Within the telemedicine‐hybrid model, effectiveness was higher with telemedicine than in‐person care (99.2% versus 98.1%, *P* < 0.001). Acceptability of telemedicine was high (96% satisfied) and 80% reported a future preference for telemedicine.

**Conclusions:**

A telemedicine‐hybrid model for medical abortion that includes no‐test telemedicine and treatment without an ultrasound is effective, safe, acceptable and improves access to care.

**Tweetable abstract:**

Compelling evidence from 52 142 women shows no‐test telemedicine abortion is safe, effective and improves care.

## Tweetable abstract

Compelling evidence from 52 142 women shows no‐test telemedicine abortion is safe, effective and improves care.

This article includes Author Insights, a video abstract available at https://vimeo.com/bjog/authorinsights16668

## Graphical abstract

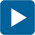

## Introduction

Improved access to care for induced abortion (termination of pregnancy) would deliver significant advantages for both healthcare systems and the women who use them. There is clear evidence that restricting access to abortion does not reduce abortion rates, it simply makes the procedure less safe.^1^, ^2^ Improving access is likely to benefit those who are most vulnerable,^3^ especially in resource‐poor settings or where care has to be self‐funded. In its 2019 guideline on abortion care, the National Institute for Health and Care Excellence (NICE) stated that improving access to abortion was a key priority.^4^

Telemedicine, the use of information and communication technologies to improve patient outcomes by increasing access to care and medical information,^5^ has been noted to decrease costs and increase convenience and safety.^6^ It is an established service delivery model for abortion care in many settings^7^ and it is recommended to improve access.^4^ The COVID‐19 pandemic required urgent action to ensure delivery of essential health services, with the Royal College of Obstetricians and Gynaecologists (RCOG) publishing guidelines to safeguard abortion care in the UK.^8^ These guidelines profoundly changed the way medical such care is delivered in Great Britain. Prior to the emergence of COVID‐19, all patients seeking medical abortion were required to attend in‐person to receive an ultrasound scan and have mifepristone administered within the clinic. Under the new guidelines, consultations were encouraged to take place by telephone or video call; an ultrasound scan was required only if indicated. By 30 March 2020, all the governments in Great Britain had issued emergency legal orders to allow mifepristone to be used at home along with misoprostol up to 10 weeks’ gestation.^9^, ^10^, ^11^ These approvals permitted abortion providers to implement a fully telemedical service delivery model, including ‘no test medical abortion’ and direct‐to‐patient delivery of abortifacient medications.

Great Britain’s new pathway for no‐test medical abortion is unusual among existing models because it is fully remote: no clinic visit, tests or ultrasound scan are performed and both mifepristone and misoprostol are delivered by mail or collected from a clinic for use at home. This new service model thus presents an important opportunity to evaluate a potentially better way to provide medical abortion that could improve access and reduce the barriers posed by in‐person care.^12^ We examined and compared the effectiveness, safety and acceptability of medical abortion provided up to 10 weeks’ gestation before and after the widespread implementation of no‐test telemedicine.

## Methods

### Population and cohorts

The study population comprised all patients who accessed an early medical abortion (EMA) at the three largest abortion providers in England – British Pregnancy Advisory Service (BPAS), MSI Reproductive Choices (MSUK) and the National Unplanned Pregnancy Advisory Service (NUPAS) – 2 months before and after the service model change. Medical abortion is defined as the use of medications to terminate a pregnancy without primary surgical intervention and ‘EMA’ applies to these procedures, commonly within the first trimester.^13^ The recommended EMA regimen uses the anti‐progestogen mifepristone in an oral dose of 200 mg, then, after 24–48 hours, 800 mcg of the prostaglandin analogue misoprostol by the sublingual, vaginal or buccal route. In the UK, an additional dose of misoprostol is recommended if expulsion has not occurred 3–4 hours after the first dose.^8^, ^14^ Prior to telemedicine, patients would have returned to clinic to receive this, but in the new model the additional dose was provided in the treatment pack. Patients are advised to call the abortion provider on their 24‐hour phone service in the event of any problems, if bleeding is unexpectedly light or heavy, if pregnancy symptoms fail to resolve quickly or if a low‐sensitivity pregnancy test (1000 IU) is positive 3 weeks after using misoprostol. This information is conveyed in a variety of formats, including verbally at consultation, in writing and through on‐line resources. Women are offered self‐assessment with a low‐sensitivity pregnancy test to determine success of the abortion, with instructions to report back to the abortion provider if there are any ongoing issues or a positive test, in line with national guidelines^15^ and high quality evidence that self‐assessment is safe, effective and preferred by women.^16^

Our dataset consisted of information on EMAs extracted directly from each provider’s electronic records and included fully de‐identified patient clinical and demographic characteristics. Each providers’ clinical incident database was cross‐referenced to patients in each cohort to determine rates of unsuccessful medical abortion and adverse events. All data were extracted 6 weeks after the end of the study period to ensure the reporting of complications was as complete as possible. We also consulted with regulators and national agencies to ensure that we accounted for incident reports made directly to them. The independent regulator of all health and social care services in England, the Care Quality Commission (CQC), confirmed that all cases reported directly to them through various routes, for example, statutory notifications and the central NHS database of patient safety incident reports (the National Reporting and Learning System [NRLS] and the Strategic Executive Information System [StEIS]), were known to the providers.

**Figure 1:**
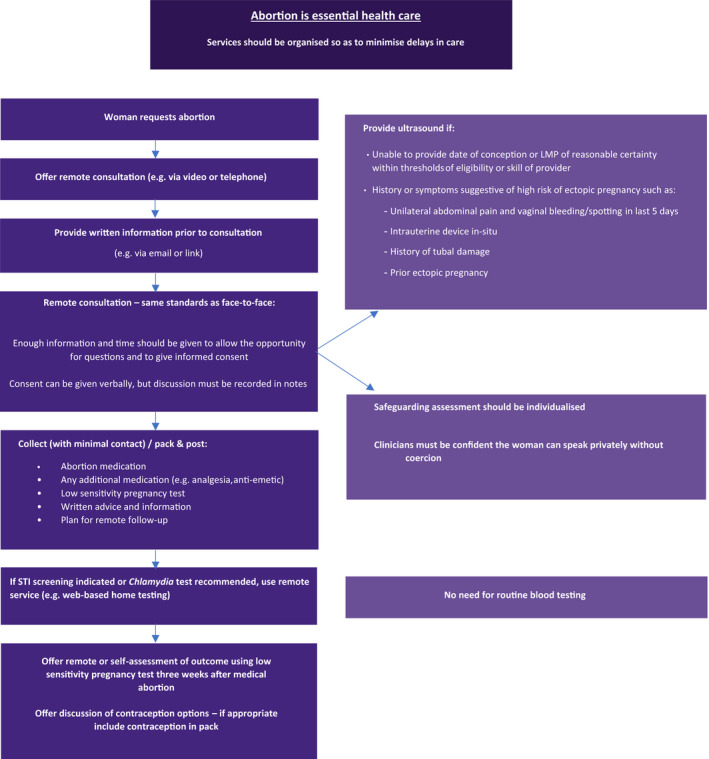
Summary of early medical abortion care management during COVID‐19 pandemic (adapted with permission from RCOG Coronavirus [COVID‐19] Infection and Abortion Care – Information for Healthcare Professionals^8^).

**Figure 2:**
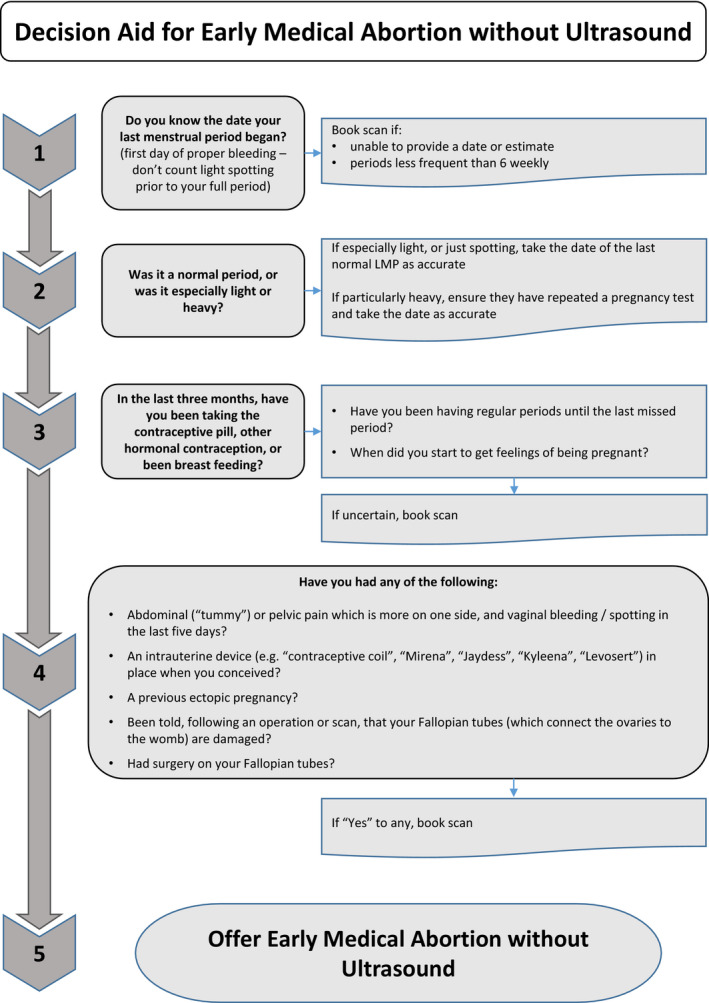
Decision aid for early medical abortion without ultrasound (adapted with permission from RCOG Coronavirus (COVID‐19) infection and abortion care – information for healthcare professionals; 2020‐06‐04‐decision‐aid‐for‐early‐medical‐abortion‐without‐ultrasound.pdf [rcog.org.uk]^17^)

Two cohorts were defined. The ‘traditional’ cohort comprises all patients having an EMA between 1 January and 1 March 2020, prior to service model change. All patients in this cohort received an in‐person assessment and an ultrasound scan, had mifepristone administered in the clinic, and were supplied with misoprostol for use at home. The ‘telemedicine‐hybrid’ cohort comprises all patients accessing an EMA between 6 April and 30 June 2020, in a 2‐month period after the service model change at each provider. Patients in this cohort were offered a consultation via phone or video call, during which an assessment of eligibility for treatment via telemedicine was made. Patients were deemed eligible for no‐test medical abortion via telemedicine if they had a low risk of ectopic pregnancy and their self‐reported last menstrual period (LMP) indicated a gestation of <10 weeks. Medications were then delivered to patients via post or were made available for collection from a clinic for use at home. Those deemed ineligible for no‐test medical abortion via telemedicine had an in‐person assessment with ultrasound as per the ‘traditional’ model. After the in‐person assessment, medications for this group were then provided from the clinic for home use. The medications provided to both groups in the hybrid cohort were the same. Providers followed organisation‐specific evidence‐based policies informed by the RCOG^8^ and associated decision aid^17^ (Figures 1 and 2) and earlier RCOG and NICE guidelines.^14^, ^15^

### Outcome measures

#### Access

Waiting time and gestation at treatment were used to assess how the two models impacted access. Waiting time was defined as the interval from first contact with the abortion provider to when medication was dispensed (either in‐person in clinic or posted). Gestation was recorded as that applicable on the date mifepristone was dispensed or provided. Analysis was of mean gestation and the proportion of abortions performed at ≤6 weeks’ gestation.

#### Effectiveness

Effectiveness was defined as the proportion of medical abortions that were successful. Success was defined according to the MARE Guidelines as successful expulsion of an intrauterine pregnancy without need for surgical intervention,^13^ using the sub‐categories as listed in Table 2.

#### Safety

Safety was defined according to the proportions of medical abortions that involved one or more significant adverse events. We defined significant adverse events as haemorrhage requiring transfusion, significant infection requiring hospital admission, major surgery and death. We also examined the incidence of ectopic pregnancy and when it was diagnosed in the care pathway. For the analysis of ectopic pregnancies, we included all abortion consultations rather than all abortions provided because some patients with suspected ectopic pregnancies were referred to Early Pregnancy Assessment Units (EPAU) and did not proceed to abortion. All patients referred for further diagnostics (e.g. serial human chorionic gonadotropin [βhCG] monitoring) but who had no further treatment are included in the ectopic pregnancy group, although a proportion of these will be pregnancy of unknown locations (PULs), which includes failed early intrauterine pregnancies. We analysed the proportion of cases where treatment was reported to have occurred at ≥10 weeks’ gestation in the telemedicine‐hybrid cohort.

#### Acceptability

Given the constraints of delivering healthcare during COVID‐19, it was not possible to follow up all patients to capture patient‐reported outcomes. Two of the providers (BPAS and MSUK) collected patient feedback during the study period. Patients were invited to provide feedback 1–3 weeks after treatment, either by telephone using a structured interview tool (MSUK)^18^ or using an online form (BPAS).^19^ We analysed data on questions reporting on satisfaction, future preference and privacy. Although the questions were similar, only the MSUK survey included questions on the privacy of teleconsultation. All contact was by a non‐clinician who had not been involved in the patient’s care.

#### Analysis

The primary analysis was to assess whether the telemedicine‐hybrid model was non‐inferior to the traditional model. We compare effectiveness and safety in the two cohorts by testing the hypotheses that the telemedicine‐hybrid model is less effective and has a higher complication rate.

We first compared patient demographic and clinical characteristics between the cohorts to assess the need to covariate‐adjust our hypothesis tests for systematic differences in the two groups that might affect outcomes of abortion. All hypothesis tests were covariate adjusted for patient age, race/ethnicity, gestational age, parity and prior abortions using logistic regression and weighted risk differences.^20^

We evaluated effectiveness by testing the alternative hypothesis that the rate of successful medical abortion in the telemedicine‐hybrid cohort is lower than in the traditional cohort using a covariate‐adjusted test of difference in proportions. We also performed a Chi‐square test to evaluate whether the distribution of unsuccessful abortion sub‐categories differed between the cohorts. We evaluated safety by testing the alternative hypothesis that significant adverse events occurred at higher rates in the telemedicine‐hybrid cohort than in the traditional cohort using a covariate‐adjusted hypothesis test for difference of proportions. We reported one‐sided *P*‐values for both of these tests. We also evaluated whether the prevalence of ectopic pregnancies managed before EMA and after EMA were different between the traditional and telemedicine‐hybrid cohorts using Chi‐square difference of proportion tests.

The secondary analysis was to compare effectiveness and safety of medical abortion for patients who received fully remote no‐test telemedicine versus in‐person care in the telemedicine‐hybrid cohort, primarily to assess whether any differences between the cohort service models were driven by one particular group. We performed covariate‐adjusted hypothesis tests under the null hypothesis of equal effectiveness and equal rates of significant adverse events in the telemedicine versus in‐person groups.

All analyses were performed using R, version 3.6.2 (R Foundation for Statistical Computing, c/o Institute for Statistics and Mathematics, Vienna, Austria). Statistical significance was defined using an alpha level of 0.05.

### Ethics approval

The study was reviewed by the Institutional Review Board (IRB) at the University of Texas at Austin, and a determination was made that the research did not meet the criteria for human subject research as defined in the Common Rule (45 CFR 46) or FDA Regulations (21 CFR 56). Each provider ensured compliance with their own internal ethics and governance systems. This study is classed as service evaluation by the NHS Health Research Authority^21^ and therefore did not require review by an NHS Research Ethics Committee (REC).^22^ The principles of the STROBE statement, and the MARE supplement, were followed.^13^ Patients were not involved in the development of the study.

## Results

In total, 52 142 medical abortions were provided during the study period: 22 158 in the traditional cohort and 29 984 in the telemedicine‐hybrid cohort. Among those that took place in the telemedicine‐hybrid cohort, 18 435 (61%) were provided via telemedicine and 11 549 (39%) in‐person. Our sample represents 85% of the total number of medical abortions performed in England and Wales during the study period.^23^ The clinical and demographic characteristics of patients in the two cohorts are described in Table 1.

**Table 1:** Clinical and demographic characteristics in the traditional and telemedicine‐hybrid cohorts (*n* = 52 142). *n* (%)

Patient characteristics	Traditional (*n* = 22 158)	Telemedicine‐hybrid (*n* = 29 984)	*P*‐value
Mean gestational age in weeks (SD)	6.4 (1.3)	6.0 (1.4)	<0.001
Gestational age at treatment*			
≤6 weeks	5582 (25.2)	11 947 (39.8)	<0.001
>6 weeks	16 576 (74.8)	18 037 (60.2)	
Mean age in years (SD)	27.8 (6.6)	28.5 (6.7)	<0.001
Ethnicity			
Asian	2038 (9.2)	2652 (8.8)	<0.001
Black	1656 (7.5)	2282 (7.6)	
Multiracial	1004 (4.5)	1361 (4.5)	
White	15 840 (71.5)	20 910 (69.7)	
Other	489 (2.2)	638 (2.1)	
Unknown	1131 (5.1)	2141 (7.1)	
Previous abortions			
0	13 098 (59.1)	16 741 (55.8)	<0.001
1+	9060 (40.9)	13 243 (44.2)	
Parity			
0	10 133 (45.7)	11 741 (39.2)	<0.001
1+	12 025 (54.3)	18 243 (60.8)	
Mean waiting time in days (SD)^*^	10.7 (19.9)	6.5 (13.5)	<0.001

* After checking for normality, these variables were non‐parametric and therefore two‐sample Wilcoxon tests were used.

### Access

Mean waiting time to treatment declined from 10.7 days (SD 19.9) in the traditional pathway to 6.5 days (SD 13.5) in the telemedicine‐hybrid cohort (*P* < 0.001). Mean gestational age at treatment also declined in the telemedicine‐hybrid cohort, resulting in 40% of abortions performed at 6 weeks’ gestation or less versus 25% in the traditional cohort (*P* < 0.001).

### Effectiveness

Rates of successful medical abortion were high under both service delivery models (Table 2) – 98.2% in the traditional cohort versus 98.8% in the telemedicine‐hybrid cohort. We found no evidence of a lower success rate with the telemedicine‐hybrid pathway (*P* > 0.999). The distribution of the different sub‐categories of unsuccessful medical abortion did not differ between the cohorts (*P* = 0.268).

**Table 2:** Comparison of effectiveness of medical abortions conducted in the traditional and telemedicine‐hybrid cohorts (*n* = 52 142). *n* (%)

Outcome	Traditional *n* = 22 158	Telemedicine‐hybrid *n* = 29 984	*P*‐value
Successful medical abortion	21 769 (98.2)	29 618 (98.8)	1.0
Unsuccessful medical abortion	389 (1.8)	366 (1.2)	
Continuing pregnancy: treated with surgical management	161 (0.7)	150 (0.5)	0.268
Continuing pregnancy: opted to continue or unknown	3 (0.01)	8 (0.03)	
Retained products treated with surgical management (ERPC)	225 (1.0)	208 (0.7)	

As explained in the methods section, the *P*‐value for successful medical abortion is the co‐variate‐adjusted *P*‐value (i.e. all differences in patient clinical and demographic characteristics, including gestational age, are controlled for) and was calculated using a hypothesis test where the null hypothesis is that the traditional cohort has the same effectiveness rate as the telemedicine‐hybrid cohort and the alternative hypothesis is that the traditional cohort has a higher effectiveness rate than the telemedicine‐hybrid cohort. The *P*‐value for unsuccessful medication abortion is the Chi‐square test of whether the distribution of types of failure differ between the cohorts.

### Safety

Significant adverse events in both cohorts were rare (Table 3). Haemorrhage requiring transfusion was reported in eight (0.04%) cases in the traditional cohort and in seven (0.02%) cases in the telemedicine‐hybrid cohort. No cases of significant infection requiring hospital admission, major surgery or death were reported. We found no evidence that significant adverse events were higher in the telemedicine‐hybrid cohort (*P* = 0.557).

**Table 3:** Comparison of significant adverse events following medical abortions conducted in the traditional and telemedicine‐hybrid cohorts (*n* = 52 142). *n* (%)

Outcome	Traditional (*n* = 22 158)	Telemedicine‐hybrid (*n* = 29 984)	*P*‐value
Haemorrhage requiring transfusion	8 (0.04)	7 (0.02)	0.557
Infection requiring hospital admission	0 (0.0)	0 (0.0)	
Major surgery	0 (0.0)	0 (0.0)	
Death	0 (0.0)	0 (0.0)	

As explained in the methods section, the *P*‐value was calculated using a hypothesis test where the null hypothesis is that the traditional cohort has the same rate of adverse events as the telemedicine‐hybrid cohort and the alternative hypothesis is that the traditional cohort has a lower rate of adverse events than the telemedicine‐hybrid cohort.

The overall incidence of ectopic pregnancy was equivalent in both cohorts – 39 (0.2%) in the traditional cohort and 49 (0.2%) in the telemedicine‐hybrid cohort, *P* = 0.796 (Table 4). The proportions managed after EMA were not significantly different between the cohorts (0.01% in the traditional pathway and 0.03% in the telemedicine‐hybrid pathway, *P* = 0.123). There were 11 cases (0.04%) in the telemedicine‐hybrid cohort where the gestational age after abortion was reported by the patient or an admitting hospital as being greater than the expected 10 weeks. In all these cases, the abortion was completed at home without additional medical complications.

**Table 4:** Significant outcomes among patients presenting for medical abortion in the traditional and telemedicine‐hybrid cohorts (*n* = 52 218). *n* (%)

Outcome	Traditional (*n* = 22 197)	Telemedicine‐hybrid (*n* = 30 021)	*P*‐value
Ectopic managed pretreatment	37 (0.17)	39 (0.13)	0.796
Ectopic managed post‐treatment	2 (0.01)	10 (0.03)	0.123
Gestational age later than expected*	0 (0.0)	11 (0.04)	N/A

The column numbers include patients who presented for an EMA but did not receive one because their ectopic pregnancy was identified pretreatment.

* The column numbers for the gestational age later than expected category are the same as those in Table 3, (all EMAs performed in the two cohorts, i.e. *n* = 52 142).

### Acceptability

Patient‐reported outcome data were available from 2453 respondents: 96% were ‘satisfied’ or ‘very satisfied’ with their care, or rated their experience as ‘good’ or ‘very good’; 80% reported that they would choose telemedicine in the future or that it was their preferred option, with 13% choosing in‐person care and the remainder being unsure. No patient reported that they were unable to consult in private using teleconsultation (*n* = 1243).

### Secondary outcome measures

Comparison of clinical outcomes for the telemedicine versus in‐person groups in the telemedicine‐hybrid cohort is shown in Tables [Link], [Link], [Link]. Rates of successful medical abortion were higher in the telemedicine group (99.2% versus 98.1%, *P* < 0.001), but rates of significant adverse outcomes were not significantly different between the two groups – three (0.02%) for telemedicine versus four (0.03%) for in‐person (*P* = 0.532).

## Discussion

### Main findings

We found that no‐test medical abortion via telemedicine without routine ultrasound up to 10 weeks’ gestation is an effective, safe and acceptable service model. Clinical outcomes with telemedicine are equivalent to in‐person care and access to abortion care is better, with both waiting times and gestational age at the time of the abortion significantly reduced. Although gestation could be influenced by different behaviour during the pandemic, resulting in earlier presentation, in most other areas of healthcare, access has been severely impacted, with waiting times increasing substantially,^24^ and so reductions in both seem relevant. Even small reductions in waiting time are significant – NICE noted that a reduction of 1 day resulted in annual savings of £1.6 million to the health services in England owing to reduced complications and fewer needing to opt for a surgical abortion.^4^ Further evidence that the new telemedicine‐hybrid model improves access comes from a study showing that the rate of women seeking abortion medication outside the formal healthcare setting was significantly reduced in the UK following its implementation.^25^ The implication is that those previously too vulnerable to attend in‐person have been able to access care through telemedicine, potentially benefitting from the safeguarding, counselling and contraceptive services provided by regulated providers.^26^

Our study confirms previous literature that medical abortion is safe and effective. Our findings of low rates of significant complications and failure are similar to those reported from other high quality studies.^27^, ^28^ The slight increase in effectiveness we observed in the group that received telemedicine – even after controlling for lower average gestational age compared with the in‐person group – may be due to the ability of patients to control better the time at which they took the medication.

The telemedicine‐hybrid model resulted in very low rates of undiagnosed ectopic pregnancy and later than expected gestations. Although the rate of ectopic pregnancy in the general population in the UK and USA is reported as 1–2%,^29^, ^30^ the rate reported among patients having a termination of pregnancy is 10 times lower,^31^ which is consistent with our findings. Ultrasound is not used to screen for ectopic pregnancy in the general population – it is only used where signs and symptoms suggest a need.^30^ Routine screening of symptom‐free women is associated with a high false‐positive rate when the prevalence of ectopic pregnancy is low, as is the case in women seeking abortion, and therefore it is unlikely there would be significant benefits.^32^ There is no clinical justification for maintaining this inconsistency in care between women wishing to continue their pregnancies and those choosing EMA.^33^, ^34^

However, given that over 200 000 people access abortion care each year in the UK alone, some will inevitably have an asymptomatic ectopic pregnancy and so will proceed with having mifepristone and misoprostol either through telemedicine or after a false‐negative scan. The essential issue for safety is that these are detected prior to causing harm rather than prior to beginning the medical abortion treatment; treatment with mifepristone and misoprostol in itself will have no effect on an underlying ectopic pregnancy. Indeed, the reduction in waiting times afforded by the telemedicine model may facilitate earlier detection than traditional pathways where women present later or are sent away to give additional time to visualise an intrauterine pregnancy on scan. Proceeding with early medical abortion without a scan may permit earlier diagnosis of a developing ectopic pregnancy owing to increased surveillance and index of suspicion, for example where there is minimal bleeding after misoprostol.^8^, ^15^

The proportion of cases where gestational age was later than expected based on LMP was low, as might have been expected given the evidence that women can determine the gestational age of their pregnancy with reasonable accuracy by LMP alone.^33^ Nevertheless, inadvertent treatment of gestations over 10 weeks is inevitable and, consistent with our findings, the consequences for most are unlikely to be medically significant.^35^ The 10‐week gestation limit in the English government’s approval order is arbitrary and is not based on evidence of safety or effectiveness. The Scottish government did not stipulate a limit, leaving the decision to the discretion of the clinician in consultation with their patient. Moreover, the reported success of self‐managed terminations of pregnancy at >12–24 weeks’ gestation is 93%, with safety similar to that expected in earlier gestations.^36^, ^37^

### Strengths and limitations

Although the study is not a clinical trial, we were able to evaluate the outcomes of both the telemedicine‐hybrid and traditional in‐person services as they operate in the real world, and we were able to adjust for key covariates. A key strength of the study is the generalisability of our findings, given that our sample included 85% of all medical abortions provided in England and Wales during the study period.

The main limitation of this study is that we were unable actively to follow up patients after their abortion. There is a potential gap in the consistency of reporting incidents, due to some complications not meeting the threshold of serious incidents, multiple routes of entry into the NHS and informal communication between the NHS and abortion providers. Although it is possible that some patients presented to other providers and a significant adverse event was not reported in our dataset, the risk management and reporting systems within the NHS are well defined, with serious incidents being routinely shared. The governing body of the NHS in England alerted all commissioners of the need to report incidents relating to telemedicine and there were review meetings of key stakeholders to ensure compliance. No additional cases were identified from regulators that were not already recorded by the providers’ clinical incident processes. More importantly, there is no reason that any under‐reporting would be systematically more likely in either cohort to introduce bias. Although patient behaviour may have been altered in the pandemic, it seems unlikely patients would not have reported problems to their provider given that there is immediate access to help via 24‐hour telephone services. It is also possible that with NHS acute services (e.g. early pregnancy units) harder to access, patients would be more likely to engage with their abortion provider first. Finally, evidence from an equivalent population to ours in Scotland gave almost identical results. This was a smaller cohort study that followed up all patients (*n* = 663) and cross‐checked NHS hospital records (reporting successful medical abortion 98%, haemorrhage requiring transfusion 0%, infection requiring hospital admission 0%, choosing telemedicine again in future 71%).^39^

### Interpretation

NICE conducted a systematic review and recommended using telemedicine to improve access to medical abortion care.^3^ Several models for using telemedicine to facilitate medical abortion have been described but most existing trials are small, and many required attendances to have medicines administered, an ultrasound scan or blood tests.^7^, ^38^ Our study is the first to assess a real‐world no‐test telemedicine abortion care pathway in a national population. This new national model demonstrates how a permissive framework for medical abortion can deliver significant quality improvements to those needing to access abortion care.

## Conclusion

This large study of 52 142 medical abortions demonstrates that incorporating no‐test telemedicine into the care pathway is not inferior to the traditional pathway where all patients are seen in person and have an ultrasound scan. There are advantages – waiting times and gestation at abortion are reduced and it is highly rated by patients. There was no evidence of worse outcomes in failure rate, haemorrhage, need for surgery or failure to detect ectopic pregnancy. In the 0.04% of cases where the abortion appeared to have been provided at over 10 weeks’ gestation, these were all completed at home without additional medical complications. Given the advantages of improving access to care, especially in vulnerable groups and in resource‐poor healthcare systems or where patients have to fund their own care, the evidence is compelling that no‐test telemedicine should become routine in the provision of abortion care.

### Disclosure of interests

All authors have completed the ICMJE uniform disclosure form at www.icmje.org/coi_disclosure.pdf and declare: no support from any organisation for the submitted work; no financial relationships with any organisations that might have an interest in the submitted work in the previous 3 years; no other relationships or activities that could appear to have influenced the submitted work beyond that described under the roles held by each individual. AA – Member of the Council of the British Society of Abortion Care Providers (BSACP); NG and JS – nothing to disclose; PL – Co‐author/committee member of national guidelines cited in this paper from RCOG and NICE; member RCOG abortion taskforce; council member of British Society of Abortion Care Providers (BSACP); Medical Director of BPAS; JL – Co‐author/committee member of national guidelines cited in this paper from RCOG and NICE; co‐chair of RCOG Abortion Taskforce and British Society of Abortion Care Providers (BSACP); primary employment includes work as termination of pregnancy care provider as a consultant gynaecologist and medical director. Completed disclosure of interests forms are available to view online as supporting information.

### Contribution to authorship

AA – Initial concept, developing study protocol, data analysis, writing first draft and subsequent revisions, verification of data. Overall responsibility for data analysis and principal investigator. Guarantor; NG – Developing study protocol, revision of draft paper. Overall responsibility for conduct of study including data collection at NUPAS; PL – Initial concept, developing study protocol, writing first draft and subsequent revisions. Overall responsibility for conduct of study including data collection at BPAS; JL – Initial concept, developing study protocol, writing first draft and subsequent revisions, co‐ordination and liaison. Overall responsibility for conduct of study including data collection at MSUK; JS – Data analysis including statistical advice, writing first draft and subsequent revisions, verification of data.

### Details of ethics approval

The study was reviewed by the Institutional Review Board (IRB) of the University of Texas at Austin and a determination was made on 21 April 2020 that the research did not meet the criteria for human subjects research as defined in the Common Rule (45 CFR 46) or FDA Regulations (21 CFR 56). Each provider ensured compliance with their own internal ethics and governance systems. The principles of the STROBE statement, and the MARE supplement, were followed.^13^

### Funding

This study was supported in part by the Eunice Kennedy Shriver National Institute of Child Health & Human Development of the National Institutes of Health (NIH) through Center Grant P2CHD042849, awarded to the Population Research Center at The University of Texas at Austin. The content of this article is solely the responsibility of the authors and does not necessarily represent the official views of the NIH.

### Acknowledgements

We would like to thank the Care Quality Commission (CQC), NHS England and NHS Improvement (NHSE & I), and the National Child Mortality Database (NCMD) for their advice and assistance in ensuring all complications reported within NHS systems have been included. We are also enormously grateful to the many people who helped to ensure access to essential healthcare for women needing an abortion not only continued, but was improved during the COVID‐19 pandemic. There are too many to list individually, but we would like to especially thank the following who not only helped to implement a complete service change within a few weeks, but also made this paper possible by ensuring the data were collected and accessible: BPAS – Rebecca Blaylock, Steve Cheung, Pam Field, Stephen Franklin, Jeanette Taylor, Katherine Whitehouse; MSUK – Cat James, Andrew Lord, Kay Newey, Abigail Storan; NUPAS – Aaron Flaherty, Linda Leach, Leanne MacCaffrey.

### Data‐sharing statement

The collated datasets, which include participant data with anonymised identifiers, are held by AA at the University of Texas. Consideration will be given to sharing this with bona fide researchers on application. The original data reside with the co‐authors’ own institutions. Although the data are de‐identified, some relate to very rare events and could therefore result in identification. Therefore data on complications, and data arising from clinical incident reports, will be subject to the same access restrictions as those of the organisation supplying it.

### Transparency statement

The authors affirm that the manuscript is an honest, accurate and transparent account of the study being reported; that no important aspects of the study have been omitted; and that any discrepancies from the study as originally planned (and, if relevant, registered) have been explained.

## Supporting information

**Table S1.** Patient clinical and demographic characteristics in the in‐person versus telemedicine groups for the telemedicine‐hybrid cohort (*n* = 29 984). Number (%).

**Table S2.** Comparison of effectiveness of medical terminations of pregnancy conducted in the in‐person versus telemedicine groups for the telemedicine‐hybrid cohort (*n* = 29 984). *n* (%).

**Table S3.** Comparison of significant adverse events following medical terminations of pregnancy in the in‐person versus telemedicine groups for the telemedicine‐hybrid cohort (*n* = 29 984). *n* (%).

**Video S1.** Author insights.

## Data Availability

Data available on request due to privacy/ethical restrictions.
