# Supplementary material for: Effectiveness, safety and acceptability of no‐test medical abortion (termination of pregnancy) provided via telemedicine: a national cohort study
Source: BJOG. 2021 Mar 24;128(9):1464–74. doi: 10.1111/1471-0528.16668 (PMC8360126; doi:10.1111/1471-0528.16668)
Supplement: Supplementary file 1 — Table S1. Patient clinical and demographic characteristics in the in‐person versus telemedicine groups for the telemedicine‐hybrid cohort (n = 29 984). Number (%). [file BJO-128-1464-s008.docx]

**Supplementary Tables to Accompany Text in the Results Section**

**Table S1: Patient clinical and demographic characteristics in the in-person vs. telemedicine groups for the telemedicine-hybrid cohort (n=29,984) [Number (%)].**

| **Patient Characteristic** | **In-Person (n=11,549)** | **Telemedicine**  **(n=18,435)** | **P-value** |
| --- | --- | --- | --- |
| **Mean gestational age in weeks (s.d.)*** | 6.2 (1.4) | 5.8 (1.3) | <0.001 |
| **Gestational age** |  |  |  |
| 6 weeks and under | 3,673 (31.8) | 8,274 (44.9) | <0.001 |
| Over 6 weeks | 7,876 (68.2) | 10,161 (55.1) |  |
| **Mean age in years (s.d.)** | 27.9 (6.6) | 28.9 (6.7) | <0.001 |
| **Ethnicity** |  |  |  |
| Asian | 1,060 (9.2) | 1,592 (8.6) | <0.001 |
| Black | 946 (8.2) | 1,336 (7.2) |  |
| Multiracial | 557 (4.8) | 804 (4.4) |  |
| White | 7,868 (68.1) | 13,042 (70.8) |  |
| Other | 336 (2.9) | 302 (1.6) |  |
| Unknown | 782 (6.8) | 1,359 (7.4) |  |
| **Previous abortions** |  |  |  |
| 0 | 6,388 (55.3) | 10,353 (56.2) | <0.001 |
| 1+ | 5,161 (44.7) | 8,082 (43.8) |  |
| **Parity** |  |  |  |
| 0 | 4,931 (42.7) | 6,810 (36.9) | <0.001 |
| 1+ | 6,618 (57.3) | 11,625 (63.1) |  |
| **Mean waiting time in days (s.d.)*** | 9.5 (20.3) | 4.7 (5.6) | <0.001 |

* = After checking for normality, these variables were non-parametric and therefore two-sample Wilcoxon tests were used.
