# Supplementary material for: Effectiveness, safety and acceptability of no‐test medical abortion (termination of pregnancy) provided via telemedicine: a national cohort study
Source: BJOG. 2021 Mar 24;128(9):1464–74. doi: 10.1111/1471-0528.16668 (PMC8360126; doi:10.1111/1471-0528.16668)
Supplement: Supplementary file 2 — Table S2. Comparison of effectiveness of medical terminations of pregnancy conducted in the in‐person versus telemedicine groups for the telemedicine‐hybrid cohort (n = 29 984). n (%). [file BJO-128-1464-s009.docx]

**Supplementary Tables to Accompany Text in the Results Section**

**Table S2: Comparison of effectiveness of medical abortions conducted in the in-person vs. telemedicine groups for the telemedicine-hybrid cohort (n=29,984) [Number (%)].**

| **Outcome** | **In-Person**  **n=11,549** | **Telemedicine**  **N=18,435** | **P-value** |
| --- | --- | --- | --- |
| **Successful medical abortion** | **11,329 (98.1)** | **18,289 (99.2)** | **<0.001** |
| **Unsuccessful medical abortion** | **220 (1.9)** | **146 (0.8)** |  |
| Continuing pregnancy: treated with surgical management | 133 (1.2) | 17 (0.09) | <0.001 |
| Continuing pregnancy: opted to continue or unknown | 1 (0.01) | 7 (0.04) |  |
| Retained products treated with surgical management (ERPC) | 86 (0.7) | 122 (0.7) |  |

Note: As explained in the Methods section, the p-value for successful medical abortion is the co-variate adjusted p-value (i.e. all differences in patient clinical and demographic characteristics, including gestational age, are controlled for) and was calculated using a hypothesis test where the null hypothesis is that the in-person has the same effectiveness rate as the telemed group and the alternative hypothesis is that the in-person group has a lower effectiveness rate than the telemedicine group. The p-value for unsuccessful medical abortion is for the chi-squared test of whether the distribution of types of failure differ between in-person and telemed groups.
