# Supplementary material for: Effectiveness, safety and acceptability of no‐test medical abortion (termination of pregnancy) provided via telemedicine: a national cohort study
Source: BJOG. 2021 Mar 24;128(9):1464–74. doi: 10.1111/1471-0528.16668 (PMC8360126; doi:10.1111/1471-0528.16668)
Supplement: Supplementary file 3 — Table S3. Comparison of significant adverse events following medical terminations of pregnancy in the in‐person versus telemedicine groups for the telemedicine‐hybrid cohort (n = 29 984). n (%). [file BJO-128-1464-s003.docx]

**Supplementary Tables to Accompany Text in the Results Section**

**Table S3: Comparison of significant adverse events following medical abortions in the in-person vs. telemedicine groups for the telemedicine-hybrid cohort (n=29,984) [Number (%)].**

| **Outcome** | **In-Person**  **n=11,549** | **Telemedicine**  **n=18,435** | **P-value** |
| --- | --- | --- | --- |
| **Haemorrhage requiring transfusion** | 4 (0.03) | 3 (0.02) | 0.532 |
| **Infection requiring hospital admission** | 0 (0.0) | 0 (0.0) |  |
| **Major surgery** | 0 (0.0) | 0 (0.0) |  |
| **Death** | 0 (0.0) | 0 (0.0) |  |

Note: As explained in the methods section, the p-value was calculated using a hypothesis test where the null hypothesis is that the in-person group has the same rate of adverse events than the telemedicine group and the alternative hypothesis is that the in-person group has a lower rate of adverse events than the telemedicine group.
